# Beyond the Point of No Return: A Discourse Analysis of Healthcare Professionals’ Perceptions of Digitally Supported Person-Centred, Integrated, and Proactive Care

**DOI:** 10.5334/ijic.6446

**Published:** 2022-10-12

**Authors:** Kari Dyb, Lisbeth Kvam

**Affiliations:** 1 Norwegian Centre for E-health Research, University Hospital of North Norway, Tromsø, Norway; 2 Department of Mental Health, Faculty of Medicine and Health Sciences, Norwegian University of Science and Technology, Trondheim, Norway

**Keywords:** digital support, person-centred care, integrated care, proactive care, discourse analysis, healthcare professionals

## Abstract

Most countries are facing a common challenge: a rise in the number of chronically ill patients and limited medical resources. The combination of digital support and the principles of person-centred, integrated, and proactive care (Digi-PIP care) services constitutes the most ambitious initiative for patients with long-term needs. While there is research on digital support, person-centred, integrated, and proactive care, the combination of these components has been less explored.

The data set consisted of 29 qualitative interviews with healthcare professionals involved in four Nordic Digi-PIP care initiatives. Building on prevailing discourses on the modernisation of healthcare, we used discourse analysis to determine how the professionals discussed their perceptions and experiences of the care transformation initiatives.

We identified four discourses illustrating that, despite challenges with adoption, the vision of Digi-PIP care was strongly embedded among participants across professions and contexts. In contrast to the discourses on their separate components, the emergent discourses on Digi-PIP care were surprisingly consistent. The new care model was found to be beneficial for patients, healthcare professionals, and society.

Digitalisation may vitalise and even catalyse person-centred, integrated, and proactive practices. To the employees involved, Digi-PIP has moved beyond the point of no return; it is the future of modern healthcare.

## Introduction

The number of patients with complex and long-term needs is increasing, and the quality of care for these patients is inadequate [1,2]. Furthermore, the link between an ageing population, patients with long-term needs and higher healthcare spending has been well documented [2,3], making this situation a growing public health concern [4].

High-quality care for patients with complex and long-term needs includes increased patient involvement; improved collaboration between professions, institutions, and levels of care; and good-quality illness prevention practices [5,6,7]. Person-centred, integrated, and proactive (PIP) care services provide promising solutions to this growing group of patients demanding better healthcare services [8,9].

Enhanced healthcare services entail higher costs. Therefore, innovative solutions are required. The principles of digitally supported person-centred, integrated, and proactive (Digi-PIP) care make up the most ambitious initiatives, according to Berntsen et al. [2]

Digitalisation and the transformation from organisation- to person-centred care (PCC) are prevailing policy discourses regarding the modernisation of healthcare provision in recent decades [10,11]. There is also a strong, common-sense belief that Digi-PIP care is a key component of sustainable healthcare [2]. However, research has failed to produce evidence promoting its acceptance and benefits. This is not necessarily due to a lack of services, but is instead caused by a reductionist scientific methodology blocking the way forward [2].

Since healthcare professionals are key in care transformation initiatives [12,13] and discourses are inherent, irreducible elements of all social phenomena and all social change [14], professional discourse on Digi-PIP care can point towards its future potential for adaption. To contribute to new knowledge about Digi-PIP care while avoiding a reductionist methodology, this paper identifies discourses on Digi-PIP care among healthcare professionals involved in four ambitious initiatives to help people with complex and long-term needs. The term ‘professional’ in this work includes nurses, physiotherapists, occupational therapists, nutritionists, and doctors working with patients. It also includes leaders, managers, and administrative staff actively involved in implementing Digi-PIP care in Norway and Denmark.

Building on prevailing discourses on the modernisation of healthcare, this paper’s research questions are as follows:

1. What are the main discourses regarding Digi-PIP care among the professionals involved in the four healthcare transformation initiatives?
2. How can the identified discourses contribute to our understanding of professionals’ visions for healthcare services in the future?

### Nordic healthcare systems and policy directions

The empirical contexts of this study are Norway and Denmark. Nordic countries are in a unique position to transform care for people with complex and long-term needs, as they operate on a Nordic welfare model [15]. In Nordic countries, equal access to high-quality healthcare services is a legal right for all citizens, and these services are public amenities that are tax-funded and involve low patient payment [16]. The movement towards PCC and the push towards digitalisation have been prioritised in politics and policy for years [17,18]. However, despite public funding policies and the equal access ideology, Nordic patients report the same challenges (e.g., poor coordination and integration) as other high-income countries [19].

### The study context

This study was part of an interdisciplinary research and implementation project called ‘Patients and Professionals in Partnership (3P)’ (2015–2021) [20]. The primary objective of 3P was to move safe multidisciplinary care delivery from hospitals to communities and citizens’ homes while wholly supporting citizens’ self-management efforts. The project included four empirical sites with the purpose of radically redesigning care delivery for patients with long-term and complex needs. The four sites were autonomous initiatives with independent funding and management that followed a project logic with a launch and end date.

Sites 1, 2 and 3 provided Digi-PIP care for patients with chronic obstructive pulmonary disease (COPD) living at home. The sites provided nurse-led telemedicine services through remote monitoring of vital COPD indicators and video conferencing with patients at home.

Site 1 was located in Denmark. It had been operating for a few years and was organised as an integrated part of municipal healthcare services. The site provided care for approximately 90 COPD patients. In addition to the municipal staff composed mainly of nurses and therapists, the site employed two e-doctors who provided care exclusively through electronic tools.

Site 2 was on the west coast of Norway, which had also been operational for a few years. The telemedicine service was in a local hospital and provided Digi-PIP care for approximately 50 COPD patients recently discharged from the hospital or a collaborating university hospital. The site employed two nurses who provided care exclusively through electronic tools, in addition to the existing hospital staff.

Site 3 was located in Southern Norway and was in its start-up phase. The telemedicine service was hosted by a small municipal nursing home, with two nurses providing remote care for five COPD patients as part of their regular duties. The service was connected to two local hospitals, and all patients had recently been discharged from these hospitals. The typical patient lived at home but was already enrolled in municipal homecare services before getting Digi-PIP care.

Site 4 was located in North Norway. It had been running for several years, employed approximately 15–20 staff, and included about 400 elderly and frail patients with multiple or chronic conditions at risk of (re)hospitalisation. The care transformation model emphasised the need for municipal and hospital services to work as an interdisciplinary team with the patient as the core team member. The staff had access to both municipal and hospital electronic health records. The approach towards patient-facing technologies was explorative and elective, and the staff were voluntarily testing commercially available digital technologies.

## Theory and Methods

Digi-PIP care seeks to improve care and lower the risk for clinical flaws that are a burden to individuals, healthcare organisations, and society. However, Digi-PIP care initiatives have not been broadly studied. We know little about their potential for implementation, benefits, or risks. Most qualitative studies on the digitalisation of health have been based on socio-technical or socio-medical approaches, exploring specific technologies in various contexts [21]. We study discourses on Digi-PIP care to identify and interpret patterns of meaning about the new care models across professions and contexts.

### Theory

Discourse analysis explores how text and talk are central to everyday life and contribute to the constitution of social reality [22]. It is a methodology used to understand how people discuss and approach the society or parts of the society. The subject matter is the language in use. It investigates how people make sense of a situation and helps to interpret and identify patterns of meaning [23]. Discourse can be considered a social practice and is an inherent and irreducible element or facet of all social phenomena and all social changes. Furthermore, discourses are situated and constructed within a particular context, yet always interlinked with each other and with other discourses present in time and space [14].

To our knowledge, there are no previously established discourses on Digi-PIP care. However, analyses of the first component, digitalisation, have reported conflicting discourses minimally engaged with one another’s arguments in academic, policy, service, commercial and lay texts on telehealth and telecare for chronic illnesses [24]. As such, the introduction of telehealth and telecare is hampered by different assumptions, values, and world views held by stakeholders [24].

The second component, PCC, has been a prevailing discourse in healthcare for several decades, contrasting with the bio-medical paradigm in which patients are seen as passive recipients of medical interventions [10]. It argues that patients are persons whose health, illness, well-being, hopes, and needs are intertwined with their environment. Thus, health and illness are understood from biopsychosocial perspectives [25]. PCC discourses promote getting to know the person behind the illness and facilitating care decisions aligned with a person’s values [26].

Studies on the combination of digitalisation and PCC highlight policy discourses arguing for digitalisation as an enabler of PCC [27]. In these discourses, technology facilitates the relationships between professionals and patients by providing sufficient information, patient engagement, and mutual feedback [28]. Yet, they argue that patients’ needs must be identified and designed into technology-enabled services and not vice versa.

Perspectives on the third component, integration, have been extensively discussed in this journal and include definitions based on the person, the health system, whole systems, and decision makers [29]. The journal also discusses combinations of components, such as PCC combined with integration, or integration combined with proactive care. This resonates with elements of the Digi-PIP care model.

### Data collection

The authors visited each site from 2016 to 2018. The data set consists of 29 interviews with professionals from each site. Most interviews were individual, but a few group interviews involved two or more participants. All interviews were conducted in a Scandinavian language, and all participants were familiar with the 3P project. The interviews were between 60 and 90 minutes long and took place at the participants’ workplaces. The interviews were audio recorded, and all participants signed an informed consent form. We started each interview by asking the participants to tell us their own stories about the Digi-PIP care initiatives. The question commonly prompted chronological narratives that lasted between 20–30 minutes. We interfered as little as possible, only to check for misinterpretations.

In the next phase of the interview, we used the implementation frameworks to guide the interview and to ensure that well-known dimensions of the implementation processes were covered. These frameworks were Normalization process theory (NPT) and Nonadoption, abandonment, scale-up, spread, and sustainability (NASSS) [30,31]. This paper originates from the 3P project and received ethical approval from the Regional Committee for Medical and Health Research Ethics North, nr.017/1084/REK nord.

### Discourse analysis

Inspired by Fairclough [14], we explored the discourse literature and theoretical perspectives we found most relevant to Digi-PIP care, such as digitalisation, PCC, and integrated and proactive care [10,24,26,27,28,32]. We shaped the key findings from this literature into a framework that we used (Table 1) for the data analysis. We then went back and forth between the framework and the empirical data to inform and contest the discourses presented in the Result section and in the Discussion section.

**Table 1:** Discourses related to analysing Digi-PIP care initiatives.


Digitalisation	[24]	Conflicting discourses in academic, policy, service, commercial and lay texts

Person centred care (PCC)	[10,25,26]	Prevailing discourse in health for decades, biopsychosocial perspectives on health and illness

Integrated	IJIC, https://www.ijic.org/	Variety of definitions, based on the person, the health system, whole systems, and decision makers

Proactive	IJIC, https://www.ijic.org/	Often discussed in combination with PCC or integration

Digitalisation and PCC	[27,28]	Policy discourses arguing for digitalisation as an enabler of PCC


We transcribed the audio recordings and reviewed the transcripts several times. We focused on how participants talked about Digi-PIP care, looking especially for similarities and variations between sites and professions. The authors coded the transcripts separately. We also flagged sections of the transcripts to maintain the contextuality of the codes.

During discussions, we became aware that professionals across professions and healthcare contexts consistently embraced Digi-PIP, despite contested and conflicting discourses on the components of Digi-PIP care in the literature and reports on local challenges and concerns in the empirical data. We discussed validity issues related to the positive utterances and decided to reread the interview data. During the reread, we looked for blind spots, keeping established perspectives on the components of Digi-PIP care at the forefront of the analysis (see Table 1). We also presented our preliminary results at 3P project meetings to obtain responses from the study participants and fellow researchers. Although they supported our initial ‘positive’ analysis, we developed a deeper understanding of some nuances in the transcripts. For example, we understood that utterances presented as afterthoughts could be of significance.

We continued going back and forth between the empirical data and the theoretical framework. This work revealed intertextual elements related to the professionals’ perceptions of digitalisation and identified a few utterances indicating scepticism towards extensive digitalisation. To report the professionals’ overall trust in Digi-PIP care as an inevitable part of modern healthcare, but also their concern towards extensive digitalisation, we named the first discourse ‘Beyond the point of no return, although…’.

The back-and-forth reading revealed that the professionals’ utterances could be linked to several empirical and theoretical topics, and we used spreadsheets to organise the excerpts into themes. We identified three additional discourses that we interpreted as sub-discourses, reporting why the professionals embraced Digi-PIP care.

The first sub-discourse, ‘Beneficial for the patient, although…’ came from transcript portions where the professionals talked about how and why Digi-PIP care enhanced health for patients with chronic and long-term conditions. For example, they described how Digi-PIP care improved patients’ experiences of safety through easy digital access to healthcare services and personnel. The intertextuality in this discourse relates to concerns about ‘cold technology’ replacing ‘warm hands’ and human caregivers.

The second sub-discourse, ‘Beneficial for the healthcare professional, although…’ emerged from parts where professionals described their own satisfaction with the new care practices, such as how they got to know the patients better and how interdisciplinary teamwork was fulfilling. Scepticism was demonstrated through concerns about, for example, recruitment of skilled nurses and challenges with persuading colleagues about the benefits of new practices.

The third sub-discourse, ‘Beneficial for the society, although…’ relates to how the professionals discussed society’s emerging need to face challenges in healthcare delivery. Although the intertextual elements were not that visible within this discourse, we found afterthoughts questioning technology’s position in society.

## Results

### Central discourse: Beyond the point of no return, although…

All professionals discussed Digi-PIP as inevitable to the future of modern health care. One said:

“It is unethical not to use technology and to be person-centred.” (Participant 1)

The participant concluded by emphasising that, in addition to delivering accurate and high-quality care to patients with complex and long-term needs, digital technology and proactive care would reduce healthcare costs. Another said:

“This is the future; it is inevitable.” (Participant 2)

Despite optimism, most participants said that replacing ‘the fragmented service delivery of today’ or the current ‘silo organisation of healthcare’ with Digi-PIP care was challenging. Concepts such as ‘pioneers’ and ‘lone riders’ were used to describe the professionals’ attempts to transform healthcare. One participant said:

“This is ground-breaking work; we are doing ground-breaking work!” (Participant 3)

Transforming healthcare was described as learning by doing, and staff members defined their work as challenging and high risk, with the continuous threat of shutting down the site. Still, this main discourse reflects the vision of Digi-PIP care as embedded and vital among professionals.

#### Sub-discourse 1: Beneficial for the patient, although…

The professionals claimed that providing Digi-PIP care was beneficial to patients’ health and quality of life. Concepts such as ‘self-determination’, ‘participation’, ‘involvement’, ‘quality of care’, and ‘one point of contact’ illustrate features of this sub-discourse. These concepts were found in sequences of text in which the participants talked about allowing the patients to define their own needs and to increase their involvement in health issues. Furthermore, the transcripts contained discussions about a broad spectrum of digital technologies being potentially beneficial for patients with complex and long-term needs, including iPads, fall alarms, and big data.

Providing care over distance for persons with COPD living at home was spoken of as beneficial to patients’ well-being. One participant said:

“Shame and self-consciousness are common in patients with COPD. When patients receive treatment at home, we have observed that the disease becomes less shameful and a lesser burden for patients.” (Participant 4)

According to this professional, patients seemed more at ease and experienced increased safety in a virtual care environment in comparison to hospital settings. Additionally, patients’ questions were more explicitly aimed at their individual needs in home contexts than in hospital counselling.

Professionals also highlighted the possibility of monitoring vital indicators and responding quickly to targeted medical interventions. One participant said:

“The technology enables us to detect symptoms, both improvements and relapses, at an early stage. Hence, we can intervene quickly and appropriately.” (Participant 5)

They described Digi-PIP care as beneficial for communication between patients and providers, as well as between institutions and levels of care. One participant said:

“In the beginning, it was primarily nice words. However, we learned to communicate with patients to gather their perspectives on what matters to them. Now, we proceed from this conversation. Even if the patient is hospitalised for a broken leg, the main issue might be something else. We engage in person-centred and integrated care, and the patient is involved and engaged to ensure smooth transferal between institutions and the patient’s home.” (Participant 6)

In contrast, the transcripts also contained a few statements in which professionals questioned Dig-PIP care. One participant said:

“I am a bit worried about the oldest and very sick patients. Are they in a position to take responsibility for their own health?” (Participant 8)

This statement, and others like it, were typically found in sequences where the participants reflected on the suitability of Digi-PIP care for all patients.

#### Sub-discourse 2: Beneficial for the healthcare provider, although….

The professionals also described the new ways of approaching patients as being beneficial for themselves. One said:

“I find Digi-PIP care stimulating. The interdisciplinarity is an appealing way of working.” (Participant 4)

A second participant said:

“I find it exciting, particularly the iPads; it is a new way to connect with patients. I get access to patients’ everyday environments, which is not possible in institutions where the patient accesses our environment instead. The iPads reverse this. We connect with patients in their homes, and we actually get to know them better.” (Participant 7)

A third participant said:

“The work is fulfilling. We can follow the patients over time, and we can provide accurate mentoring since we can see the patients’ specific challenges in their homes.” (Participant 8)

The discourse reflects work satisfaction, including team spirit and faith in the interdisciplinary nature of Digi-PIP care. However, it also contains statements indicating challenges. For example, one participant said:

“The work is challenging, continuously navigating between primary and secondary care.” (Participant 9)

Another said:

“We need to persuade colleagues; it is challenging.” (Participant 10)

A few participants said that Digi-PIP care is extremely demanding in terms of employment, as it requires nurses to have special skills and attitudes. Another said:

“Several doctors define their work as exclusively biomedical; they do not regard non-biomedical aspects of patient care as their responsibility. Therefore, Digi-PIP care is not among their tasks and responsibilities.” (Participant 5)

#### Sub-discourse 3: Beneficial for society, although…

Furthermore, professionals talked about society’s need for care transformation. They linked it to the changing demographics in Western societies and emphasised the need to reduce costs by transforming care as essential to the sustainability of future healthcare services. Professionals questioned society’s capability to meet what some referred to as ‘the announced silver tsunami’ and a healthcare system not prepared for the increased number of elderly citizens living longer with chronic conditions. Digi-PIP care is one central component in meeting pending societal demand. One said:

“Patients need to be activated. The system needs to move from the logic of providing treatment to the logic of proactive and preventive care.” (Participant 17)

Others said:

“Proactive citizens and preventive care—it is the way forward.” (Participant 18) or

“Technology is how it is these days.” (Participant 1)

The arguments for societal benefits were sometimes followed by afterthought, such as:

“Technology should not be used for the sake of technology.” (Participant 9) or

“Technology cannot replace human care.” (Participant 16)

## Discussion

The identified discourses illustrate that, despite challenges and concerns, the vision of Digi-PIP was strongly embedded among all the professionals in this study. Consistent faith in digitalisation, PCC, and integrated and proactive care across professions and healthcare contexts is surprising and contested by established literature [30,31], including the framework litterature [10,25,26,27,28,29]. Uncontested optimism is mainly found in policy discourses and the technology industry.

Therefore, it is important to point to the weaker, but visible, utterances that contested the main content of the discourses, which we have highlighted by including ‘although’ to each discourse. These utterances may indicate that the Nordic professionals working with chronically ill patients were, to some extent, forced to accept a larger policy discourse that is actively pushing digital technology in healthcare. Hence, the discourses may be interpreted as the pragmatic acceptance of technology’s position in society, emerging from a form of rationalising that things are generally out of professionals’ control and that acceptance is the tool to continue functioning in their day-to-day jobs.

Thus, positive language does not indicate an obedient or out-of-control feeling. This might indicate that professionals found Digi-PIP care to be an opportunity to act in accordance with their professional convictions and to practice value-driven holistic healthcare [33]. The identified discourses echo statements associated with PCC. In line with a humanistic and holistic perspective towards health and healthcare, the selected wording ‘getting to know the patients better’, ‘self-determination’, ‘negotiation’ and ‘involvement’ are at the core of practicing PCC [26].

Parallel to humanist discourses on technology [24,28], the identified discourses on Digi-PIP care illustrate that remote interactions through iPads had prompted the professionals to develop trustful partnerships with patients, provide accurate advice on medical matters, and motivate and safeguard the patients. PCC and digitalisation as prevailing discourses on the modernisation of healthcare provision correspond to research by Little [10] and Patsis [11]. However, this disagrees with Klecun [27] and Greenhalgh [24], who reported numerous and contested policy discourses of digitalisation. Additionally, it disagrees with Fox and Reeves [33], who argued that interprofessional PCC approaches reinforced the patient compliance model by shifting responsibility to patients to do the ‘right thing’ by extending the reach of medical power across other groups of professionals.

Digital technology and changed work practices can disrupt established professional relations and even trigger conflicts and power struggles among patients, professionals, and institutions [34]. The discourses illustrate that, regardless of professional or institutional belonging, professionals appreciated close interaction with patients, team spirit and the interdisciplinary nature of Digi-PIP care. Interdisciplinary and integrated care practices seemed to strengthen rather than weaken professional relations and unite individuals, professions, departments, and institutions.

The discourses indicate that Digi-PIP care served the individual patient’s biomedical and psychosocial needs. Although the need to make medicine more scientific and humanistic by including both psychological and social perspectives has been present for decades [35], implementing psychosocial elements across professions and organisations has been slow [36]. As such, professionals did not see psychosocial and biomedical approaches as contested approaches but emphasised the need for collaboration and integration when providing Digi-PIP care. The vison for the future was interdisciplinary care at the intersection of medicine, nursing, therapy, and social work.

Jacobs [34] argued that in policy discourses on transforming healthcare practices, the moral discourse on patient autonomy becomes intertwined with the instrumental discourse on healthcare budget savings. Reducing overall healthcare spending was important for the professionals in this study. Thus, instrumental discourses about cost reduction as essential for the sustainability of future healthcare services might have enhanced the professional’s faith in Digi-PIP care. Still, the identified discourses indicate that the digital technology offered opportunities to put patients at the centre and to provide both integrated and proactive services. This study demonstrates that even if digitalisation was contested, it was also seen an enabler of PIP care.

### Limitations

The 3P context was four autonomous Digi-PIP care initiatives united by the 3P project and may have coloured the participants’ statements in a positive manner. However, participants’ perceptions of the benefits for patients, themselves, and the believed gain for society at large may have been a strength and led to new and valuable knowledge through discourse analysis, even if discourse analysis is not well understood or valued by the mainstream medical community.

The authors might also have been influenced by the 3P project; other researchers may have interpreted the transcripts differently and identified other discourses. To validate our interpretations, we went back and forth between the theory and empirical data and received feedback on preliminary interpretations from fellow researchers.

## Conclusion

To the diverse group of healthcare professionals involved in four care transformation initiatives for people with complex and long-term needs in Norway and Denmark, the combination of digital support and PIP care is a promising care practice. Despite concerns and challenges, these healthcare professionals view Digi-PIP as beneficial to patients, healthcare professionals, and society at large.

The consistent belief in new care practices across professions and healthcare contexts is surprising and contested in established academic discourses. This is particularly evident in research reporting on the complexity of technological innovation in the healthcare space. The four discourses indicate a strong shared belief in Digi-PIP care, and that digital technology may vitalise and even catalyse person-centred, integrated, and proactive healthcare practices. The perceived benefits of the new care practices trumped professionals’ concerns over extensive digitalisation. The discourse analysis illustrated that, despite concerns and challenges, the vision of Digi-PIP care was strongly embedded among healthcare professionals. To them, Digi-PIP care has passed the point of no return; it is the future of modern healthcare for people with chronic and long-term needs.
